# Establishment of a New Device for Electrical Stimulation of Non-Degenerative Cartilage Cells In Vitro

**DOI:** 10.3390/ijms22010394

**Published:** 2021-01-01

**Authors:** Simone Krueger, Alexander Riess, Anika Jonitz-Heincke, Alina Weizel, Anika Seyfarth, Hermann Seitz, Rainer Bader

**Affiliations:** 1 Biomechanics and Implant Technology Research Laboratory, Department of Orthopedics, Rostock University Medical Center, 18057 Rostock, Germany; anika.jonitz-heincke@med.uni-rostock.de (A.J.-H.); anika.seyfarth@uni-rostock.de (A.S.); rainer.bader@med.uni-rostock.de (R.B.); 2 Department Life, Light & Matter, University of Rostock, 18051 Rostock, Germany; hermann.seitz@uni-rostock.de; 3 Chair of Microfluidics, Faculty of Mechanical Engineering and Marine Technology, University of Rostock, 18051 Rostock, Germany; alina.weizel@uni-rostock.de

**Keywords:** capacitively coupled electrical stimulation, cartilage regeneration, differentiation, chondrocytes

## Abstract

In cell-based therapies for cartilage lesions, the main problem is still the formation of fibrous cartilage, caused by underlying de-differentiation processes ex vivo. Biophysical stimulation is a promising approach to optimize cell-based procedures and to adapt them more closely to physiological conditions. The occurrence of mechano-electrical transduction phenomena within cartilage tissue is physiological and based on streaming and diffusion potentials. The application of exogenous electric fields can be used to mimic endogenous fields and, thus, support the differentiation of chondrocytes in vitro. For this purpose, we have developed a new device for electrical stimulation of chondrocytes, which operates on the basis of capacitive coupling of alternating electric fields. The reusable and sterilizable stimulation device allows the simultaneous use of 12 cavities with independently applicable fields using only one main supply. The first parameter settings for the stimulation of human non-degenerative chondrocytes, seeded on collagen type I elastin-based scaffolds, were derived from numerical electric field simulations. Our first results suggest that applied alternating electric fields induce chondrogenic re-differentiation at the gene and especially at the protein level of human de-differentiated chondrocytes in a frequency-dependent manner. In future studies, further parameter optimizations will be performed to improve the differentiation capacity of human cartilage cells.

## 1. Introduction

One of the main tasks of articular cartilage is the absorption of mechanical loading and reduction of friction during joint movements. Cellular and molecular structure of cartilage tissue is adapted accordingly [1,2,3]. Articular cartilage is composed of two different phases: A fluid phase (65–80%), consisting mainly of water and dissolved inorganic ions, and a solid phase (20–35%), which is composed of different types of collagens, proteoglycans, and the embedded chondrocytes. It also contains a small amount of lipids, phospholipids, non-collagenous proteins and glycoproteins [2,4,5]. Embedded chondrocytes are responsible for the synthesis and degradation of extracellular matrix (ECM) macromolecules, like collagen type (Col) II and glycosaminoglycans (GAGs). These macromolecules provide the cartilage tissue with dimensionality, elasticity, and strength to withstand mechanical loading [5,6]. Since a direct exchange of substances through vessels cannot take place, the cells are supplied with nutrients and oxygen from the synovial fluid [2] or the subchondral bone [7] by diffusion. During physical activity, the cellular supply is improved due to constant alternating phases of pressure loading and pressure relief [2]. Not only is nutrient and oxygen supply promoted by this process but also endogenous electric potentials are triggered by mechanical stress [8]. Mechano-electrical transduction phenomena within the tissue occur physiologically and are based on streaming and diffusion potentials caused by mechanical loading of the micro-structured cartilage tissue [5].

During weight-bearing and joint movement, the fluid flow provokes streaming- and diffusion-generated electric potentials by fixed ionized macromolecules within the cartilage tissue. In detail, the chains of GAGs within the proteoglycan network are carrying negative charges (carboxyl and sulfate groups), which are surrounded by dissolved counter-ions (e.g., calcium ions, sodium ions) and co-ions (e.g., chloride ions) in the interstitial water [5,9,10,11,12]. The resulting endogenous electric field is transferred to the embedded cells by macromolecules like proteoglycans, cartilage oligomeric matrix protein and others [10]. The cells can sense electrical, as well as chemical and mechanical signals from the environment via receptors and ion channels, which are located in the cell membrane [11,13]. This signal transmission works both from the cell outside to the inside and vice versa. Intracellular, a variety of cellular functions and pathways can be regulated by endogenous electric fields. This includes, among others, cell migration, proliferation, gene expression, and the release of proteins and, thus, also differentiation [13,14,15,16,17,18,19,20].

Age-related alteration, traumatic injuries or degeneration of the cartilage matrix results in a loss of the fixed micro-environment leading to disruption of the physiological electric field, which is important for tissue homeostasis [21]. To treat lesions of articular cartilage, several therapeutic approaches are used to relieve the pain within the joint and to repair the affected cartilage tissue. For this reason, treatment concepts have focused on the restoration of articular cartilage through invasive and non-invasive treatments [22,23,24]. Regenerative approaches like (matrix-associated) autologous chondrocyte implantation ((M)ACI) are widely used. Here, autologous chondrocytes which are isolated from an intact part of the patient’s articular cartilage in an initial step, are cultivated ex vivo, and re-implanted in the cartilage defect to restore the extracellular matrix of hyaline cartilage [25,26]. However, cell-based methods can lead to fibrocartilage in situ, which shows altered mechanical properties compared to physiological hyaline cartilage [24,27,28,29]. To improve the restoration of cartilage tissue, it is therefore necessary to optimize cell-based procedures and to adapt them more closely to the physiological boundary conditions. In this context, electrical stimulation is described as a possible approach to improve regeneration of the cartilage tissue [13,16,30,31].

Although therapeutic devices for electrical stimulation are entering the clinical market [32], there are only few studies dealing with electrical stimulation of cartilaginous cells [11,13,16,31,33]. Most of the described in vivo and in vitro approaches are based on pulsed electromagnetic fields (PEMF). Here, data from clinical trials suggest that PEMF is able to improve clinical scores and function of osteoarthritic patients [34]. In vitro, it was found that PEMF increases the proteoglycan release of alginate-encapsulated chondrocytes [35] and in osteoarthritic cartilage tissue [36]. In addition to PEMF, other approaches for the application of electric fields in vitro, like direct and capacitive coupling, are described in the literature. Using capacitive coupling, many disadvantages can be excluded compared to direct coupling, such as the formation of cytotoxic compounds like hydrogen peroxide or electrochemical reactions that can lead to pH changes [32,37]. Brighton et al. developed an experimental setup for electrical stimulation in vitro based on capacitively coupled electric fields (2 V/m at 60 kHz). They found that applied alternating electrical fields showed an increased anabolic effect by a concomitant decrease in matrix catabolism [11,16,31]. In previous stimulation experiments we were able to demonstrate a pro-chondrogenic effect on the differentiation capacity of human chondrocytes using capacitively coupled electric fields [38]. However, the experimental setup which we used in previous studies did not allow extensive comparative analyses of different stimulation parameter. Therefore, we have conceptualized and developed a new stimulation device for application of capacitively coupled electrical fields in vitro. In the present paper, we introduce the stimulation device, in particular the generated cell chamber, control and signal unit, as well as the user interface. In addition, initial stimulation parameter analyses were carried out with respect to the chondrogenic re-differentiation potential of human de-differentiated chondrocytes in dependency of different alternating electric fields and frequencies. 

## 2. Results

### 2.1. Stimulation Device

**Figure 1:**
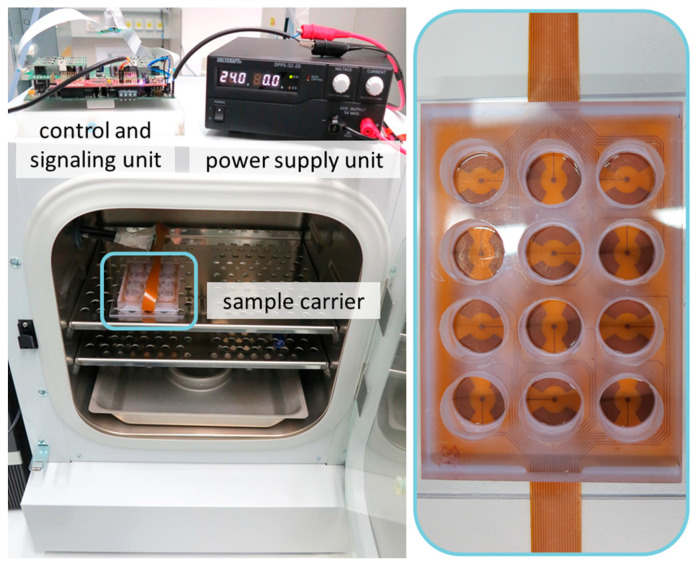
Overview of the stimulation device with control and signaling unit, sample carrier (detailed image; right) which is coupled to the electrodes and power supply unit.

The device has been designed to operate, once configured, as an automatized electrical stimulation system. Its core components are the software, the control and signaling unit, and the sample carrier (cell chamber) coupled to the electrodes (Figure 1). The stimulation device enables 12 independent configurable signals within 12 wells. The modular design allows a variable implementation of the stimulation system. While the sample carrier (cell chamber) must be placed exclusively inside the incubator and the power supply exclusively outside, the control unit can be positioned as required. For use of the control unit inside the incubator, the housing has been three-dimensionally (3D) printed for sealing against the humidified atmosphere. In this application, problems with the heat generated by the electronics can occur. For this case the external placement is provided. Here, only the signal lines of the stimulation are led into the incubator and connected to the sample carrier.

#### 2.1.1. Control Software and User Interface

The software solution includes a user interface and firmware for the microcontroller. The user interface is used to schedule the stimulation tasks. The type, start, and duration of each stimulation can be defined here. Alternatively, it is possible to import already defined stimulation cycles. After definition of the tasks, a timetable is generated and sorted chronologically. After triggering the start command, the instructions for the respective stimulation cycle are transmitted to the microcontroller via a serial interface (UART). The microcontroller converts the information into the corresponding electrical stimulation.

#### 2.1.2. Control and Signal Unit

The control unit is based on the C2000^TM^ LaunchpadXL (Texas Instruments, Dallas, TX, USA). It has been extended with self-developed circuit boards to connect the signal generators of the electrical stimulation. A socket board is implemented to accommodate the Launchpad. It provides the power supply for the components and relays the signals. The signal generator is an in-house developed circuit based on the AD9833 (Analog Devices, Inc., Norwood, MA, USA). The signal is transmitted to the electrodes via an operational amplifier with differential output. Voltages of up to 42 V peak-to-peak (V_pp_) and a maximum frequency of 2 MHz can be realized. The system contains twelve independent signal generators of this type. For calibration, input voltage, control voltage, and output voltage were measured and the amplification factor as a function of the control voltage was determined. The values determined for all signal generators were averaged, resulting in a relative deviation of the output voltage from the setpoint of 3% (data not shown).

#### 2.1.3. Sample Carrier

**Figure 2:**
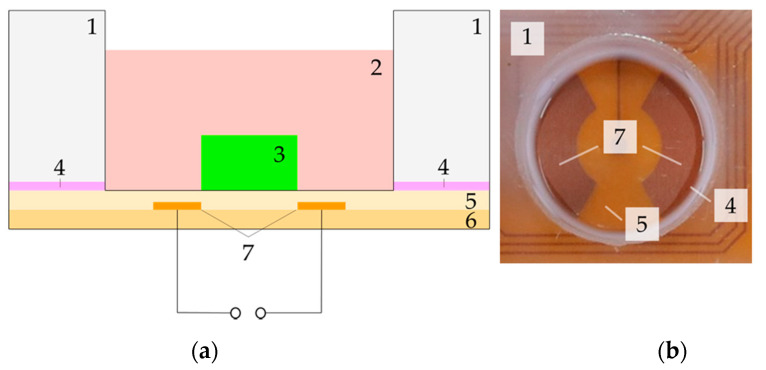

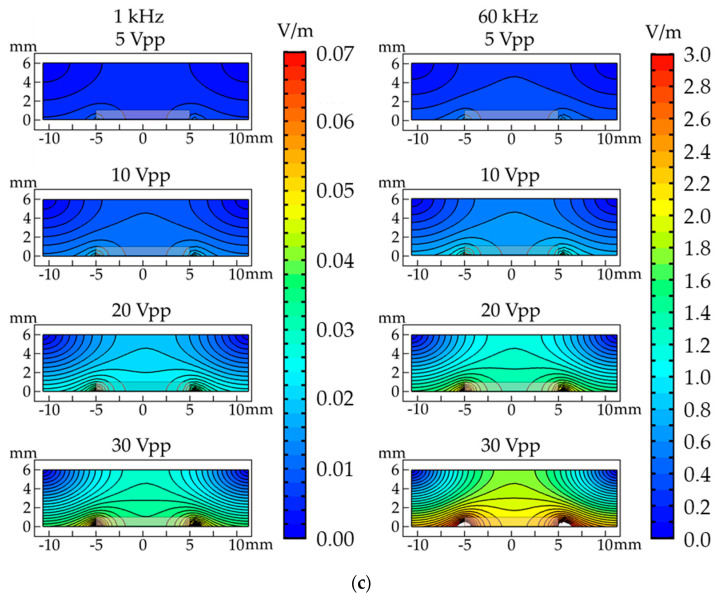
Sectional view of one well: Schematic view with (1) polycarbonate shell, (2) cell culture medium, (3) scaffold, (4) silicone-caoutchouc-adhesive, (5) 25 µm polyimide insulation layer, (6) 25 µm polyimide base layer, (7) electrodes (**a**). Real picture of one well without cell culture medium and scaffold (**b**). Electric field distributions in one well (yz-plane) for different parameter combinations (**c**).

Figure 2 shows the cross-sectional view (a), a real picture (b) and the electric field distribution (c) of one well. The sample carrier (cell chamber) consists of a polycarbonate plate (1) into which 12 cavities have been inserted by drilling with subsequent reaming. On one side of the plate, the flexible printed circuit board with the electrode array consisting of 25 µm polyimide layer with enclosed copper electrodes (5–7) is fixed by means of silicone-caoutchouc-adhesive (Troll Factory Rainer Habekost e.K., Riede, Germany) (4). The scaffold (e.g., collagen-based matrix or hydrogel) (3) is directly attached to the flexible printed circuit board and surrounded by the cell culture medium (2). Depending on the scaffold material, it can be fixed with some adhesive to ensure that it remains in place during stimulation. The cavity has a diameter of 21 mm and a height of 16 mm. The maximum height of the scaffold is limited by the required overhang of the medium. To prevent the ingress of contaminants, the entire sample carrier is covered with a 3D-printed lid with small gaps at its edges to allow gas exchange with the environment. All components of the device can be sterilized by heat or ethanol and are, thus, reusable. The electric field distribution of one well was simulated for all stimulation parameters in the yz-plane view. In the center of the electrodes, where the scaffolds are placed, the electric field amplitude is heterogeneously distributed. The resulting field, which affects human chondrocytes in 1 mm height, is either approximately 2.0 to 2.5 V/m estimated for an input voltage of 30 V_pp_ and a frequency of 60 kHz or 0.032–0.040 V/m for 30 V_pp_ and a frequency of 1 kHz. Due to the linearity of the system, the electric fields for other input voltages can be easily deduced by scaling the result.

### 2.2. Biocompatibility Testing of System Components

To ensure that no cytotoxic substances are released from components of the electrical stimulation system, an indirect cytotoxic test in conformity with the DIN EN ISO 10993-5 was performed. For this purpose, the sample carrier (cell chamber) was filled with cell culture medium and incubated under standard culture conditions for five days. The medium was removed and incubated on human chondrocytes over a period of one, two, and five days. Afterwards, cellular viability has been determined via measuring metabolic activity and live/dead staining.

**Figure 3:**
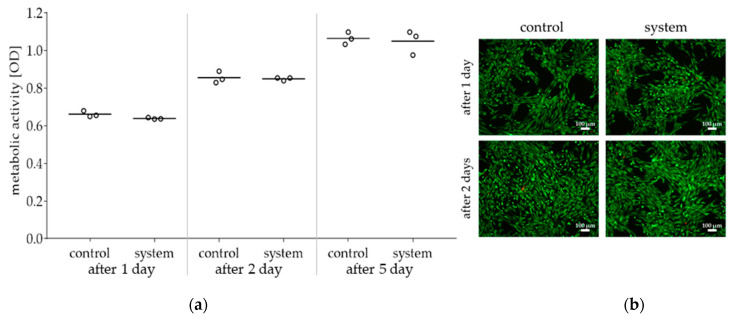
Viability of human chondrocytes following incubation for one, two or five days with conditioned medium (whole stimulation device): (**a**) Metabolic activity of chondrocytes treated with conditioned medium and corresponding control were determined via water-soluble tetrazolium salt (WST-1) assay. Data are illustrated as mean including mean values of measuring points (*n* = 3). Statistical analysis was performed with two-way ANOVA. (**b**) Live/dead-staining of chondrocytes after incubation with control medium or with conditioned medium; living cells (green), dead cells (red); the scale bar represents 100 µm.

A possible cytotoxicity of the stimulation device was not found. The metabolic activity of human chondrocytes does not deviate from the corresponding control (Figure 3a). In addition, metabolic activity increased in a time-dependent manner (*p* < 0.0001). 

Live/dead staining revealed no difference between exposed and non-exposed cells after one and two days (Figure 3b). After five days, a confluent cell layer was detected for both cell cultures (data not shown). 

### 2.3. Investigation of Stimulation Parameter

The influence of different stimulation parameters on cell activity and chondrogenic differentiation was investigated with human chondrocytes derived from non-degenerative cartilage tissue cultivated on collagen elastin scaffolds. Unstimulated cells served as controls. All cell culture experiments were done in humidified atmosphere at 5% CO_2_ and 5% O_2_. Based on literature data and our own previous research we have chosen the frequencies 1 kHz [38,39] and 60 kHz [11,16,31] for initial parameter analyses. These frequencies were combined with voltages of 5 V_pp_, 10 V_pp_, 20 V_pp_, and 30 V_pp_. In preliminary tests, maximum applicable voltage of 42 V_pp_ combined with 60 kHz showed no significant changes in cellular activity and chondrogenic differentiation and was, therefore, not integrated into the presented test series (data not shown). In addition to frequency and voltage, the stimulation duration also has an impact on cell response. Therefore, we used previously described stimulation duration of three times 45 min per day for a period of seven days [38,39]. Following electrical stimulation, we have determined metabolic activity by using WST-1 assay. Chondrogenic differentiation capacity was evaluated by gene expression analyses of the de-differentiation marker collagen type I (*Col1A1*) as well as the re-differentiation markers collagen type II (*Col2A1*) and SRY-box (*Sox*) 9. Additionally, the release of Col I and Col II propeptides was determined in supernatants of stimulated and unstimulated cells. 

Table 1 contains an overview of all achieved results. Regarding metabolic activity, no significant differences were observed after electrical stimulation. Although no significant differences were detected in *Col1A1* gene expression, a frequency-dependent trend for downregulation was noticed. This was in line with Col I propeptide data, where a frequency-dependent reduction of the release could be observed; significant for the combination of 1 kHz with 5 V_pp_ (*p* = 0.0149) and 20 V_pp_ (*p* = 0.0119) compared to 60 kHz and for 1 kHz with 10 V_pp_ (*p* = 0.0473) compared to unstimulated controls. While we were not able to determine *Col2A1* gene expression, release of Col II propeptide could be detected in both unstimulated and stimulated cells. However, no significant differences in protein contents were detectable. In addition, no significant differences were found regarding *Sox9* gene expression. However, similar to *Col1A1* gene expression, a frequency-dependent trend was observed at low voltages. 

## 3. Discussion

Electrical stimulation is successfully used for bone regeneration both in vitro and in vivo [32]. Additionally, electrical stimulation also appears to be suitable for the treatment of cartilage lesions and is therefore a good alternative to conventional treatment approaches [13,30], because it can improve the quality of re-implanted chondrocytes [11,16,17,31].

According to Vaca-González et al., several stimulation devices were used for investigations of electrical stimulation of chondrocytes or cartilage tissue [13]. The devices differ mainly in their construction: e.g., in the arrangement of the electrodes, the distance between electrodes and the resulting electric fields [13]. Due to these differences, it is difficult to compare various studies [40]. Brighton and co-workers are one of the most cited studies in the field of electrical stimulation of chondrocytes and cartilage tissue [14,41,42,43]. Their in vitro stimulation systems consisted of different kinds of Petri dishes. Metallic electrodes were placed below and on top of the dishes, which leads to a capacitive coupling of electric field into the cell or tissue culture. By modification of the used Petri dishes, a reduction of the distance between the electrodes was realized, resulting in improved dielectric material properties. This, in turn, led to reduction of applied voltages at constant field strengths [14,41,42,43]. The homogenous distributed field strength of 20 mV/cm (2 V/m) with the use of a frequency of 60 kHz was found to be the most suitable for differentiation of chondrocytes and cartilage tissue in the used stimulation system [11,15,16,31]. In detail, in studies of Brighton et al. a significant upregulation of cartilage matrix components aggrecan and collagen type II after stimulation with an electric field of 2 V/m in bovine [15] and in human osteoarthritic cartilage tissue samples [16] could be shown.

The combination of alternating voltage and frequency influences resulting electric field strength. With the input voltage of 30 V_pp_ and a frequency of 60 kHz, it is possible to achieve similar electric field strengths (2.0 to 2.5 V/m; according to our simulation results) in our stimulation device as in the setup of Brighton et al. [11,16,31]. We achieve this despite the use of low voltages, which are beneficial when handling voltage-carrying equipment. Analogue to Brighton et al. and other groups, we use sinusoidal signals [14,15,31,33], but our configuration additionally allows triangular voltages. However, it must be noted that the field alignment in our device differs from the Brighton et al. system. This also leads to the effect that air does not play a role as a possible additional insulator.

Due to the design and arrangement of the electrodes, our new stimulation system generates a heterogeneous electric field. In detail, the electrodes are located semi-circular below the cell-seeded scaffold without being covered. Therefore, field strengths decrease from the electrodes to the well centre and from the electrode interface to medium surface. Hence, we expect to be able to mimic the physiological electric fields distribution in cartilage tissue with this electrode arrangement; as the occurrence of cartilage matrix components is also inhomogeneous. The proteoglycan concentration increases from the cartilage surface to the subchondral zone, while water content and Col II concentration behave oppositely. The density of cells decreases from the non-load- to the load-bearing region of the articular cartilage. Since proteoglycans are unequally distributed in the tissue, fixed charges in it are not uniformly distributed. This leads to assumption that resulting endogenous electric fields occur heterogeneously in cartilage tissue [5]. Additionally, capacitively-coupled stimulation systems offer several advantages for electrical stimulation of human cells or tissues. There is no direct cell- or electrolyte-electrode interaction, thus preventing electro-chemical reactions of the electrode material. Moreover, cell damage by formed noxious substances due to redox reactions at electrolyte-electrode interface can be avoided by using this kind of electric field stimulation [37]. Furthermore, the arrangement of the electrodes in our system enables the future combination with mechanical stimulation through compression and shear stress by using 3D scaffolds [44]. In addition, our system also allows the use of other electrode arrangements, which would result in a change of the field direction. 

An important aspect for cell culture is that cultivation is performed in a defined environment. Therefore, our device is designed for use in an incubator with humidified atmosphere and a defined temperature. Additionally, a hypoxic atmosphere can be provided, which mimics physiological conditions in cartilage tissue [39,45]. In addition to the shown biocompatibility, our new stimulation device also addresses the aspect of sustainability, as all components can be sterilized by heat or ethanol and are, therefore, reusable. 

**Figure 4:**
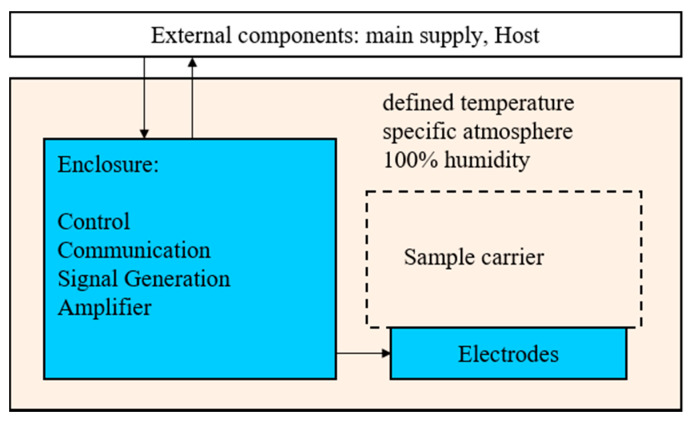
Interaction between the subsystems.

For our initial parameter study we stimulated twelve cell-seeded Col I elastin-based scaffolds independently, by using only one main supply (Figure 4). In this context, our own previous studies were disadvantaged by systems as the number of necessary function generators was limited [38,39,46]. Other partially commercially available systems would be unfavorable for our approach. On the one hand, this is due to the use of direct electrical stimulation [47,48,49,50]. On the other hand, the use of additional, external generators is necessary, which limits parallel parameter optimization [49,50,51]. 

The results of our initial parameter study revealed a frequency-dependent impact on chondrogenic re-differentiation capacity of human chondrocytes. In contrast to Brighton et al. [15,16] and own previous study [38] we could not detect *Col2A1* gene expression and there was no change neither in *Sox9* gene expression nor in Col II propeptide release compared to unstimulated controls after electrical stimulation for seven days. At this point, however, it must be noted that the time period of seven days is relatively short to significantly induce the synthesis of pro-chondrogenic factors. However, trends are emerging, which will be clarified by extended stimulation duration in subsequent test series. Data from Brighton et al. also show that stimulation exceeding a period of seven days increased effect on proteoglycan concentration [15]. In cell-based therapies like (M)ACI the de-differentiation of chondrocytes during ex vivo expansion is still the main problem [52,53,54]. After re-implantation these altered cells begin to form fibrous cartilage [55], which main component is Col I [29]. With regard to de-differentiation processes, a reduced Col I protein release could be detected after stimulation with 1 kHz. This trend is also evident in *Col1A1* gene expression at low input voltages. Although we used Col I-based scaffolds in our study, our results may show that, primarily, the applied frequency leads to a decrease in de-differentiation processes. This would be a first important step to improve the chondrogenic differentiation capacity of the cells. Subsequently, it should be analyzed in long-term stimulation experiments if electrical stimulation leads further to an accumulation of hyaline matrix components.

In order to achieve significantly enhanced cartilage regeneration in situ, optimal parameters for electrical stimulation must be identified. A combination of different stimulation durations, frequencies and voltages could be a suitable tool to enhance chondrogenic differentiation. Corresponding results have already been demonstrated by Brighton et al. [14,42,56] and emphasize the importance of adjusted stimulation parameters. In a previous study with another stimulation chamber we have seen a voltage- and frequency-dependent re-differentiation of stimulated human osteoarthritic and non-degenerative chondrocytes [38]. As our new stimulation device has an automatic stimulation control, a sequence of different combinations of stimulation parameters is possible and will be tested in subsequent experiments. We were able to identify slight differences with the first parameter analysis; further cultivation conditions have to be adapted in order to achieve significant changes in the chondrogenic differentiation capacity. In addition to the total stimulation time, the type of 3D matrix is a main challenge for reaching optimal stimulation results. Moreover, it is also conceivable that the additional use of non-Col I materials could lead to improved physiological properties. Although Col I materials are mainly used for cartilage repair, these matrices do not reflect the physiological condition and probably interfere with hyaline chondrogenic differentiation [2,4]. So far, there are only a few Col II-based materials that would correspond more to the natural surroundings of the chondrocytes [57,58]. Furthermore, materials influence the applied electric fields by conductivity properties, so that a material should be used that has similar conductivity properties to hyaline cartilage matrix. A limitation of our electric field simulation is based on the assumption that the used scaffold has same conductivity properties as the cell culture medium, caused by complete absorption of the medium. Therefore, no influence on field distribution is assumed. However, since these properties are an important influencing variable, these should be investigated with suitable instruments and included in numerical field simulations. Furthermore, we assume that the electric field of one well does not affect the electric field distribution in the scaffold of adjacent wells. Moreover, edge effects would be negligible due to the positioning of the scaffolds in the center of two circular ring segment-shaped electrodes at the bottom of the wells. Experimental validation of the electric field will have to be part of future investigations. 

For cartilage repair expanded autologous chondrocytes are commonly used [26]. However, it must be notices that these cells have two disadvantageous characteristics. They are not stable in ex vivo monolayer culture and change their phenotype to a prechondrogenic mesenchymal-like one, and they are rarely present in donor tissue [26,59]. Therefore, the physiological quality of the cells must be regained before they can be re-implanted into weak cartilage tissue. Electrical stimulation with alternating fields seems to be a suitable tool to induce chondrogenic re-differentiation. [13,16,30,31]. For a prospective practical application, it would be conceivable that donor-derived cells would be extracted as is already done for (M)ACI and expanded ex vivo in our stimulation device. Obtained chondrocytes for re-implantation would have an improved re-differentiation status compared to unstimulated chondrocytes, which may reduce the risk of fibrocartilage formation. This assumption has to be proven during long-term and in vitro studies. In the current version of our stimulation device, the applied parameter combinations are saved as log files. However, with regard to a clinical application, the measurement, automatic documentation and any necessary automatic adaption of the resulted electric fields would have to be enabled in the future. In addition, in the literature it is described that a co-cultivation of chondrocytes and mesenchymal stem cells could also lead to improved cartilage regeneration [60,61]. Since mesenchymal stem cells also react to electric fields with chondrogenesis [62,63], a combination of electrical stimulation with mechanical loading, a suitable 3D scaffold close to cartilage properties as well as the use of co-cultivated cells could lead to an optimal basis for re-implantation of cultivated autologous chondrocytes into the cartilage defect. 

In conclusion, we could establish a new device for electrical stimulation of chondrocytes in vitro, which operates on the basis of capacitive coupling of alternating electric fields. Our initial results show that the applied electrical fields lead to reduction of Col I protein expression in human de-differentiated chondrocytes in a frequency-dependent manner. This, in turn, could lead to improved quality of regenerated cartilage tissue after re-implantation of stimulated cells into injured tissue. Nevertheless, electrical stimulation period and scaffold material have to be further optimized. In the future, a combination of electrical and mechanical stimulation within the in vitro device and use of more articular cartilage tissue-like scaffolds may further improve the outcome by mimicking the physiological environment of articular cartilage tissue.

## 4. Materials and Methods 

### 4.1. Concept of Electrical Stimulation Device

The electrical stimulation device was planned for use within an incubator to ensure stable atmospheric and temperature conditions around the specimen (Figure 4). Therefore, all electronic components had to be enclosed and sealed to avoid any unwanted interchange of the atmosphere, especially humidity. This separation was achieved by an enclosure sealed with a silicone adhesive (Troll Factory Rainer Habekost e.K., Riede, Germany). All mechanical parts were 3D printed with a material based on polylactic acid (PLA) (PLA Plus, Filamentworld, Neu-Ulm, Germany). Within the enclosure a microcontroller board (LaunchPadXL, Texas Instruments) takes place as a low-cost control and communication solution. 

It was extended by a printed circuit board for signal routing, power supplies and connectors for the stimulation stage. All voltage measurements for calibration were performed with a PicoScope 2205MSO oscilloscope (Picotech, Cambridgeshire, UK). Further, a sample carrier (cell chamber) with tight coupling to the electrodes as well as the electrodes themselves was needed. Programming of the microcontroller was done with CodeComposerStudio (TexasInstruments, TX, USA) using C/C++. The development of printed circuit boards and the electrodes has been performed using EAGLE 9.6.1 (Autodesk, Inc., San Rafael, CA, USA). Ease of use was guaranteed by the deployment of a software solution created in MATLAB^®^ 2018a (The MathWorks, Inc., Natick, MA, USA) providing a graphical user interface. The software includes the definition of the stimulation tasks and a scheduler for their execution. All mechanical constructions were conducted with PTC Creo Parametric 5.0 (Parametric Technology GmbH, Unterschleissheim, Germany).

### 4.2. Numerical Simulations of Electric Fields Distribution

**Figure 5:**
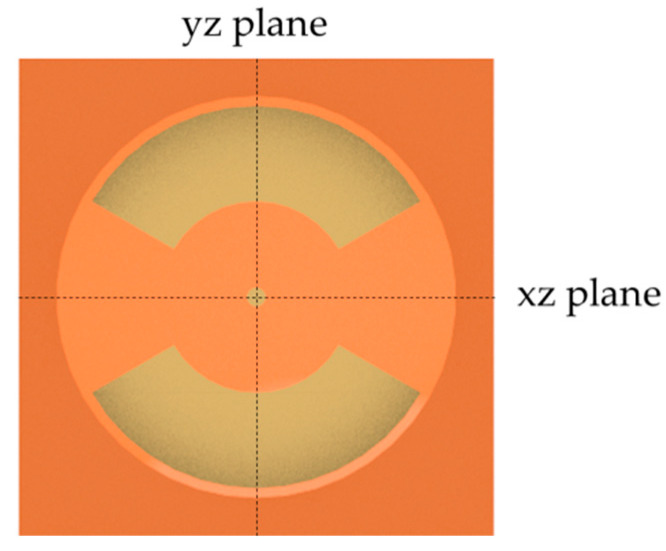
Schematic view of one well with labeled yz and xz plane-directions for electric field simulation.

The electrical field distribution inside the new stimulation device was numerically modeled with COMSOL Multiphysics 5.4 (Comsol Multiphysics GmbH, Berlin, Germany). A schematic view of the setup is presented in Figure 2a and a schematic view of one well with labeled plane-direction for electric field simulation is mentioned in Figure 5. 

The thin polyimide insulation layer over the electrodes was defined by the ‘contact impedance’ boundary condition with a thickness of 25 µm. The electrodes were assumed as ground and terminal with time-varying input voltages. The simulations were carried out in the frequency domain as an electro-quasi-static problem. All outer boundaries of the device were modeled as electrical insulators. The used material parameters in the model are summarized in Table 2. All simulations were performed with an Intel Xeon E5-2643 (3.3 GHz, two sockets with eight cores) workstation with 128 GB RAM under Windows 10 (64-bit).

### 4.3. Biocompatibiltiy Testing of Stimulation Device Components

Biocompatibility tests of system components were carried out under consideration of all used materials in direct or medium contact. Therefore, commercially available human chondrocytes isolated from non-degenerative articular cartilage tissue of the knee joint (NHAC-kn; male donor: 30 years, CC-2550, LONZA Walkersville Inc., Walkersville, MD, USA) were expanded and cryopreserved at passage 3. For cell cultivation, chondrocytes were cultured in Dulbecco’s Modified Eagle Medium (DMEM; Gibco^®^; Thermo Fisher Scientific Inc., Waltham, MA, USA) with 10% fetal bovine serum (FBS; Pan Biotech, Aidenbach, Germany), 1% penicillin/streptomycin (Pen/Strep; Thermo Fisher Scientific, Inc., Waltham, MA, USA), 1% Amphotericin B (Biochrom GmbH, Berlin, Germany), and 50 µg/mL ascorbic acid (Sigma-Aldrich, Merck KGaA, Darmstadt, Germany) in a 175 cm^2^ cell culture flask (Thermo Fisher Scientific, Inc., Waltham, MA, USA) at 37 °C in a humidified atmosphere containing 5% CO_2_ and 5% O_2_ (hypoxia). When confluence reached 90% the cells were enzymatically detached and frozen in aliquots. For biocompatibility test of the stimulation device in its entirety, a medium conditioning experiment was carried out. In detail, 3 mL of DMEM with 10% FBS were added into each well of the stimulation device and incubated at hypoxic cell culture conditions for five days. 

For subsequent analysis of the conditioned media, cells were thawed and cultured in 75 cm^2^ cell culture flasks (Thermo Fisher Scientific, Inc., Waltham, MA, USA) at the same hypoxic cell culture conditions as previously described. Afterwards, cells were detached and seeded at passage 4 as triplicates in a 24-well plate (Corning, Inc., Corning, NY, USA) with a density of 100,000 cells per well (50,000 cells/cm^2^) allowing adherence in DMEM with 10% FBS containing 1% Pen/Strep, 1% Amphotericin B and ascorbic acid (50 µg/mL) at cell culture conditions for 24 h. Afterwards, the medium was fully replaced by the conditioned medium to which ascorbic acid (50 µg/mL) was added. 

For the evaluation of a possible cytotoxicity effect of the used materials the metabolic activity of the cells was detected after one, two, and five days via water-soluble tetrazolium salt (WST-1)-assay (Takara Bio Inc., Kusatsu, Japan). This assay is based on the reduction of the tetrazolium salt by mitochondrial dehydrogenase to formazan, which produce a photometrically measurable color change. The optical density of this color change was detected and quantified at a wavelength of 450 nm and a reference wavelength of 630 nm. Therefore, cells were incubated with diluted WST-1-reagent (dilution 1:10 with DMEM with 10% FBS) for 30 min at hypoxic cell culture conditions. Afterwards, the absorbance was measured as triplicates of 100 µL in a 96-well plate (Thermo Fisher Scientific, Inc., Waltham, MA, USA) using Dynex Opsys MR^TM^ Microplate Reader (Dynex Technologies Inc., Chantilly, VA, USA). 

After washing once with phosphate-buffered saline (PBS; Biochrom AG, Berlin, Germany) cells were stained with Live/Dead-dye (Thermo Fisher Scientific, Inc., Waltham, MA, USA). In detail, 4 µM Ethidium homodimer 1 and 2 µM Calcein AM were solved in PBS. The contained dye Calcein AM (Ex/Em 494/515 nm) is retained by living cells, therefore these cells fluorescent green after excitation. Ethidium homodimer 1 (binding to nucleic acids: Ex/Em 495/635 nm) accesses exclusively cells with damaged membrane. Dead cells are not able to exclude the dye and fluorescent red. The stained samples were imaged with the Nikon Eclipse 120 fluorescence microscope (Nikon Instruments, Tokyo, Japan) by using FITC- and G-2A-filter on matching positions, respectively. Afterwards, images of living and dead cells were overlaid by using the GNU Image Manipulation Program (GIMP, Version 2.8.4.). 

### 4.4. Initial Parameter Analyses

An advantage of the newly constructed stimulation device is the possibility of stimulating 12 samples in parallel. This opportunity was used to study the effect of different parameter combinations (voltage and frequency) on the biological response of human chondrocytes. 

For investigations of stimulation parameters, human chondrocytes (LONZA) were thawed and cultured in 25 cm^2^ cell culture flasks at the hypoxic cell culture conditions. At subconfluence, cells were detached and 50,000 chondrocytes were seeded at passage 4 on three-dimensional collagen elastin scaffolds (Matriderm; MedSkin Solutions Dr. Suwelack AG, Billerbeck, Germany), which were 10 mm in diameter and attached in the middle of each electrode area with biocompatible silicone adhesive (Korasilon paste, Kurt Obermeier GmbH and Co. KG, Bad Berleburg, Germany). After an initial adherence time of 30 min, each well was filled up with 2 mL DMEM containing 1% Pen/Strep, 1% Amphotericin B, 1% Insulin-Transferrin-Selen (ITS+^TM^ Premix, BD Biosciences, Franklin Lakes, NJ, USA), 50 µg/mL ascorbic acid, 100 nM dexamethasone (Sigma-Aldrich, Merck KGaA, Darmstadt, Germany), and 50 ng/mL insulin-like growth factor (IGF)-1 (R&D Systems, Minneapolis, MN, USA), as well as 50 ng/mL transforming growth factor (TGF)-β1 (Peprotech, Hamburg, Germany). After three days of incubation under hypoxic cell culture conditions the medium was exchanged by medium without growth factors and the electrical stimulation was started by the MATLAB configuration and maintained three times for 45 min per day for seven days. Each parameter combination was examined in triplicate and the experiment was performed twice. Thus, six samples per parameter combination and 16 related controls were available for further cell biological investigations.

The following parameter combinations were examined (Table 3):

#### 4.4.1. Cellular Activity

After electrical stimulation the metabolic activity was determined as described before (Section 2.1.3); with the exception that scaffolds were transferred to a 24-well plate and incubated for 45 min with diluted WST-1-reagent (dilution 1:10 with DMEM without FBS) under cell culture conditions. 

#### 4.4.2. Gene Expression Analyses

The gene expression of *Col1A1*, *Col2A1*, and *Sox9* was examined as described in [38]. Briefly, scaffolds were enzymatically digested with collagenase A for approximately 10 min. Afterwards total RNA was isolated using peqGOLD Total RNA Kit (VWR International GmbH, Darmstadt, Germany) according to manufacturer’s recommendations. For subsequent transcription into cDNA with High Capacity cDNA Reverse Transcription Kit (Applied Biosystems, Forster City, CA, USA), 150 ng RNA was used. To determine the expression rates of de-differentiation and re-differentiation-associated genes, the cDNA of the stimulated and unstimulated cells was used for semi-quantitative real-time polymerase chain reaction (qRT-PCR). Using the following conditions: 95 °C for the initial 2 min and 39 cycles of 5 sec at 95 °C and 25 sec at 60 °C, qPCR was performed in a qTOWER 2.0 (Analytik Jena, Jena, Germany). The used primer sequences are mentioned in Table 4. 

#### 4.4.3. Protein Synthesis 

The release of Col I and Col II was determined through the measurement of their propeptides in supernatant of electrically stimulated and unstimulated cells as previously described in [38].

Briefly, for measuring the amount of type I C-terminal collagen propeptide (CICP), the MicroVue CICP ELISA (Quidel, San Diego, CA, USA) was used according to manufacturer’s instructions. Collagen type II ELISA (IBEX, Montréal, QC, Canada) was used to measure the concentration of type II C-terminal propeptide (CIICP). 

All protein data were normalized to total protein content, which was measured using a Qubit^®^ Protein Assay (Thermo Fischer Scientific Inc., Waltham, MA, USA). 

### 4.5. Statistics and Data Illustration

GraphPad Prism v.7.05 (GraphPad Software, Inc., San Diego, CA, USA) was used for data analysis and illustration. The results for biocompatibility testing are illustrated as the mean including measuring points (mean values) and for metabolic activity, gene, and protein expression as the mean and standard deviation. To detect outliers the ROUT outlier test was performed. 

For biocompatibility tests, human chondrocytes were used in triplicates. The underlying statistics were performed with the mean values of the repeated measured optical density data. Samples, treated with conditioned medium, were compared to associated controls by a two-way ANOVA test to examine time- and treatment-dependent effects. 

To investigate the effect of electrical stimulation six samples per parameter combination and 16 related controls were examined. The underlying statistics for metabolic activity were performed with the mean values of the repeated measured optical density data. The gene expression data are shown as 2^(−ΔΔCt)^ in percent. For data analysis a cycle of threshold (Ct) limit of 28 was used. The relative expression of each gene compared to the housekeeping gene β-Actin was calculated using the equation: ΔCt = Ct (target gene) − Ct (β-Actin). The relative amount of target mRNA in unstimulated and stimulated cells was calculated using 2^(−ΔΔCt)^ with ΔΔCt (treatment/control) = ΔCt (stimulated/control) − ΔCt (mean value of unstimulated controls related to experiment). To allow the statistical comparison to the unstimulated controls, the underlying statistical analysis was performed with the ΔΔCt values. For the statistical analysis of protein data, values of investigated protein amounts normalized to total protein content were used. 

Normal distribution of results after stimulation was tested with Shapiro–Wilk. All data passed the Shapiro–Wilk test and were compared by ordinary one-way ANOVA with Bonferroni’s multiple comparison test. The significance level was set to a *p*-value less than 0.05. Further details of statistical tests are indicated in the results section and the figure/table legends.

## Figures and Tables

**Table 1:** Overview of metabolic activity, gene expression and protein release of de-differentiation and re-differentiation markers following electrical stimulation (three times 45 min per day). After seven days metabolic activity of human chondrocytes seeded on collagen elastin scaffolds was determined via WST-1 assay; accumulated supernatants were collected and cells were lysed. Afterwards, RNA was isolated and reverse transcribed. Gene expression analyses were performed by semiquantitative real-time polymerase chain reaction (qRT-PCR). Supernatants were used for type I C-terminal collagen propetide (CICP) and type II C-terminal collagen propeptide (CIICP) ELISA. Data are shown as mean and standard deviation (*n* ≥ 5). Significant differences between groups were calculated with ordinary one-way ANOVA with Bonferroni´s multiple comparison test (* *p* < 0.05; stimulated vs. unstimulated control; ^#^ *p* < 0.05, 1 kHz vs. 60 kHz).

		Metabolic Activity (OD)	*Col1A1* (%)	CICP Protein (ng/mg)	*Sox9* (%)	CIICP Protein (ng/mg)
**control**		0.457 ± 0.123	101.3 ± 15.9	766.1 ± 184.2	101.1 ± 16.2	105.1 ± 29.7
**5 V_pp_**	1 kHz	0.416 ± 0.124	90.7 ± 16.9	485.7 ± 47.5 ^#^	88.8 ± 7.4	86.5 ± 36.2
	60 kHz	0.427 ± 0.044	114.0 ± 17.8	867.0 ± 340.3	108.0 ± 18.6	79.2 ± 21.7
**10 V_pp_**	1 kHz	0.342 ± 0.097	85.7 ± 3.3	481.3 ± 75.1 *	90.2 ± 25.1	91.3 ± 25.1
	60 kHz	0.401 ± 0.052	99.0 ± 13.5	752.7 ± 173.0	98.8 ± 14.9	107.8 ± 28.6
**20 V_pp_**	1 kHz	0.324 ± 0.116	92.7 ± 23.3	492.4 ± 47.2 ^#^	98.2 ± 10.8	82.0 ± 29.3
	60 kHz	0.383 ± 0.038	101.0 ± 16.6	881.0 ± 243.4	109.2 ± 24.9	78.9 ± 32.5
**30 V_pp_**	1 kHz	0.357 ± 0.107	92.2 ± 21.9	566.5 ± 83.9	104.0 ± 11.5	96.7 ± 17.5
	60 kHz	0.450 ± 0.063	89.2 ± 15.2	899.1 ± 125.2	93.2 ± 21.8	93.3 ± 17.9

**Table 2:** Material parameters in the simulation model.

Material	Conductivity [S/m] σ	Relative Permittivity ε
Air	10 × 10^−15^	1
Cell medium	1.5	80
Electrodes (copper)	5.998 × 10^7^	1
Insulation layer (polyimide)	1 × 10^−10^	3.4

**Table 3:** Overview of used stimulation parameter combinations.

Applied Frequency (kHz)	Applied Voltage (V_pp_)	Electric Field Affecting Cells (Approximately) (V/m)
1	5	0.004–0.006
1	10	0.010–0.012
1	20	0.022–0.026
1	30	0.032–0.040
60	5	0.3–0.4
60	10	0.6–0.8
60	20	1.3–1.6
60	30	2.0–2.5

**Table 4:** Overview of used primers for qRT-PCR.

Gene		Primer Sequence	Description/Function
β-Actin (ACTB)	forward	5′-CTTCCTGGGCATGGAGTC-3′	Housekeeping gene
	reverse	5′-AGCACTGTGTTGGCGTACAG-3′	
Collagen I (*Col1A1*)	forward	5′-ACGAAGACATCCCACCAATC-3′	De-differentiation marker
	reverse	5′-ACGAAGACATCCCACCAATC-3′	
Collagen II (*Col2A1*)	forward	5′-AATGGTGGCTTCCATTCAG-3′	Main macromolecule of the ECM of cartilaginous tissue
	reverse	5′-GTGATGTTCTGGGAGCCTTC-3′	
SRY-box 9 (*Sox9*)	forward	5′-AGTACCCGCACCTGCACAAC-3′	Transcriptional factor mediating chondrocytes phenotype and cartilage homeostasis
	reverse	5′-CGCTTCTCGCTCTCGTTCAG-3′	

## Data Availability

The data presented in this study are available on request from the corresponding authors.
